# A Review of Wearable Technologies for Elderly Care that Can Accurately Track Indoor Position, Recognize Physical Activities and Monitor Vital Signs in Real Time

**DOI:** 10.3390/s17020341

**Published:** 2017-02-10

**Authors:** Zhihua Wang, Zhaochu Yang, Tao Dong

**Affiliations:** 1 Institute of Applied Micro-Nano Science and Technology, Chongqing Technology and Business University, Chongqing 400067, China; Wang1072020701@hotmail.com; 2 College of Mechatronic Engineering and Automation, National University of Defense Technology, Changsha 410073, China; 3 Institute for Microsystems (IMS), Faculty of Technology and Maritime Science, University College of Southeast Norway (HSN), Horten 3184, Norway

**Keywords:** elderly care, wearable technologies, indoor positioning, human activity recognition, vital sign monitoring

## Abstract

Rapid growth of the aged population has caused an immense increase in the demand for healthcare services. Generally, the elderly are more prone to health problems compared to other age groups. With effective monitoring and alarm systems, the adverse effects of unpredictable events such as sudden illnesses, falls, and so on can be ameliorated to some extent. Recently, advances in wearable and sensor technologies have improved the prospects of these service systems for assisting elderly people. In this article, we review state-of-the-art wearable technologies that can be used for elderly care. These technologies are categorized into three types: indoor positioning, activity recognition and real time vital sign monitoring. Positioning is the process of accurate localization and is particularly important for elderly people so that they can be found in a timely manner. Activity recognition not only helps ensure that sudden events (e.g., falls) will raise alarms but also functions as a feasible way to guide people’s activities so that they avoid dangerous behaviors. Since most elderly people suffer from age-related problems, some vital signs that can be monitored comfortably and continuously via existing techniques are also summarized. Finally, we discussed a series of considerations and future trends with regard to the construction of “smart clothing” system.

## 1. Introduction

Aging populations are bringing significant challenges to societies everywhere [1]. Worldwide increases in elderly populations are demanding more healthcare services, including those of hospitals and nursing homes. For elderly people unable to take care of themselves, it is critical for a nurse or family member to pay extra attention to them during their daily care. Ordinarily, the costs of elderly care in hospitals, nursing homes or by employing professional nurses are very high. These approaches may bring financial pressure for the families with aged people, even worse the elderly with chronic conditions. A prospective solution that can reduce these costs is to care in private homes with the help of wearable technologies [2]. Fortunately, the advent and advance of wearable technologies have opened a door to develop feasible devices for elderly care.

Currently, smartwatch, smartphone and smart clothing are the mainstream products embedded wearable technologies with care functions. All of them have attractive advantages for delivering health information. For instance, smartphones are ubiquitously carried by people everywhere and every day; besides the big enough screen of smartphones can behave as a great avenue for Human-Computer-Interaction (HCI). However, the limited number of sensors and the locations where sensors are placed restricted smartphones’ functions. Functions that need skin contact monitoring are difficult to be realized by smartphones. Smartwatches, a networked computer with an array of sensors, can realize continual connection to the skin to monitor physical signals. Moreover, smartwatches are body mounted, with a standard, fixed location. This means we do not need to fix sensor locations. Nevertheless, smartphones also suffer from the constraints such as limited sensor quantities and locations. Another gimmick product that can behave as “e-health” systems for elderly care is smart clothing, which incorporates technological capabilities into existing wear [3]. A superior advantage of smart clothing is that this platform can embed more sensors to realize diverse function than smartwatch or smartphone. At present, several smart clothing solutions have been reported already. For example, smart shirts by Heddoko^TM^ (Montreal, Canada) collect full-body bio-mechanical data that can be viewed in real time or saved for later playback via a cellphone app [4]. Similarly, health related smart shirts that can measure heart and respiratory rates and the intensity of wearers’ workouts have reportedly been developed by Hexoskin, Cityzen Sciences, Ralph Lauren Polo, and Athos [5]. All these smart shirts are designed to monitor the status of various human physiological properties while their wearers are exercising. In addition, some companies (i.e., Mimo Baby [6] and Owlet Baby Care [7]) have developed novel smart clothes for babies that can track sleep status, breathing, body position, and orientation and forward the information to a monitoring application.

Quite a lot similar smart clothes have been designed that can be used for elderly care. Some of these can recognize the physical activity and monitor the physiological vital signs of the elderly; some are capable of early disease detection (e.g., heart attacks or Parkinson’s disease). However, traditional smart clothes cannot track precise position. Image that when an elderly people suddenly fell down, and very serious, he or she may require a prompt response from doctors and nurses to avoid additional injuries [8], the first thing is to locate the elderly people quickly. In this review, considering the specially requirements of elderly care, we extended the current smart clothing concept and presented the wearable technologies for the design of elderly care systems include methods for precise positioning, tracking physical activity, and monitoring vital signs in real time.

Positioning involves accurate localization of elderly people, including in both indoor and outdoor locations. Outdoor positioning is performed outside buildings and indoor positioning is performed inside buildings (e.g., houses, hospitals, and malls) [9]. There are several well established and widely used navigation systems for outdoor positioning, such as the Global Positioning System (GPS), the Global Navigation Satellite System (GLONASS), Galileo Positioning System (GALILEO) and BeiDou Navigation Satellite System (BDS). All of these are satellite-based systems for which ground-based sensors rely on signals from at least four satellites to estimate user coordinates. These technologies are currently accurate to approximately several meters for outdoor scenarios [10]; however, they cannot be used to determine precise indoor locations because of the significant attenuation of satellite signals in buildings. The indoor positioning errors of satellite-based systems are currently unacceptably large. Hence, the existing satellite-based positioning technologies can meet the demands of elderly care only for outdoor scenarios.

Based on the explanation above, this review emphasizes precise indoor positioning technologies. For elderly care scenarios, precise indoor positioning should work continuously in real-time. In recent decades, numerous indoor positioning approaches, such as Bluetooth, WiFi/WLAN, radio frequency identification (RFID), ultra-wideband (UWB), have been developed; however, these vary greatly in terms of their resolution, coverage, precision, technology, scalability, robustness and security [10,11,12]. Considering the special demands of elderly care, solutions can be selected or developed by using existing technologies to address the problems. For example, through fusing two or more types of the existing technologies using proper algorithm, the performance of that will be improved in a certain extent.

For human activity recognition (HAR), several approaches have been proposed. The current up to date researches on this topic can be mainly divided into two categories: vision-based recognition and sensor-based recognition [13,14]. For vision-based HAR, systems require a two-dimensional (2D) video recorder or a low-cost integrated depth sensor (such as the sensors and cameras in a Kinect™) [15]. Human movements are recognized from Red, Green, and Blue (RGB) data in conjunction with depth data. Considering the likelihood that many elderly people not only reside indoors but also spend time outside, vision-based HAR is unsuitable for elderly care because it is both difficult and too expensive to install cameras in all the places where elderly people are active. Moreover, the recognition accuracy of such systems decreases in outdoor environments because of variable lighting and other disturbances [14,16]. Therefore, vision-based systems are limited to specific environments in which such visual disturbances can be controlled. Thus, this review emphasizes sensor-based HAR. With the development of Micro Electro Mechanical System (MEMS) technologies, wearable sensors integrated with inertial, acceleration and magnetic sensors are becoming increasingly less expensive, smaller, and lighter. Currently, MEMS sensors are widely applied for human activities recognition, behavior classification and human activity monitoring domains [13,14,17].

As people become older and older, the majority of elderly persons have some typical old-age-related problems such as high blood pressure, high blood cholesterol, cerebral thrombosis, and so on. Therefore, it is necessary to develop real-time physiological status monitoring systems (e.g., electrocardiogram (ECG), body temperature, blood pressure, blood oxygen concentration, and so forth) to ensure their quality of life, safety and well-being. These data can be transmitted to a smartphone or personal computer (PC) by a cable or wireless signals [18]. On one hand, these data can be used to monitor health status without requiring the intervention of a doctor or other caregiver. When the data signify an obvious problem from which an elderly person may be at risk, the monitoring system can immediately trigger an alarm that results in a rapid intervention by medical or civil protection services personnel. On the other hand, these data can also be collected by authorized entities or governmental organizations to evaluate the global or national health status in order for the promotion of reasonable policies. Thus, such health monitoring systems can help to reduce the costs of healthcare by forecasting disease risks and can improve the quality of life and safety of the elderly by helping to manage existing conditions.

**Figure 1:**
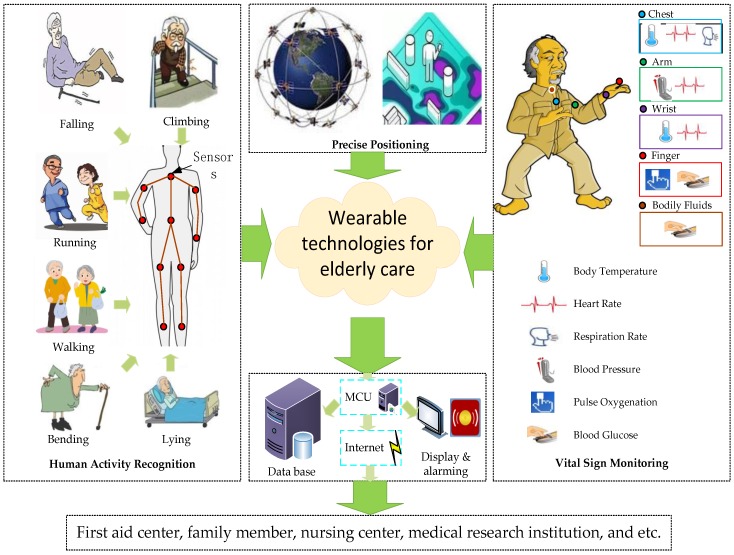
Schematic of functions for elderly care, including precise indoor positioning, physical activity tracking and real-time monitoring of vital signs.

Figure 1 illustrates a schematic drawing of functions that we presented in this review for the design of wearable elderly care systems. Technologies primarily serve to acquire accurate indoor positioning, physical activity tracking and physiological status monitoring data of the elderly in real time. Thereinto, a precise indoor positioning sensor network with wireless/wired technologies must be developed to track people’s positions in real time. In addition, a software system that includes modules for data processing, feature extraction, physical activity recognition, and intelligent decision making must be developed to support HAR. Moreover, the biomechanical sensors that can monitor the physiological parameters are increasingly promising for integration into a prototype for elderly care. This prototype for elderly care, configured with multiple sensors, will be incorporated into clothing worn by the elderly. Hence, in this review, we summarize the existing knowledge and state-of-the-art technologies that can be used in the design of the wearable elderly care systems. The main contents of this article is as follows:

- Investigate and summarize the state-of-the-art technologies to achieve precise indoor positioning based on wireless/wired communication;
- Compare up to date physical activity tracking approaches that incorporate multiple sensors into sensing nodes wearable by people;
- Present some flexible and wearable medical devices that have been demonstrated with applications in vital signs monitoring, utilizing flexible, stretchable, and lightweight materials for fabricating the biosensors.

To identity literature for this review, the following terms: “elderly care”, ”wearable technology”, ”smart clothing”, “smart home”, “positioning”, ”indoor positioning”, “human activities recognition”, “vital sign monitoring” are used for searching references. As the aim of this paper is to provide an overview of wearable technologies for elderly care, we identified wearable technologies that can meet the demands or can be improved to satisfy the needs of elderly care scenarios, including those have been used in smartphones and smartwatches for positioning, HAR, and vital sign monitoring, since the majority of these technologies can be used for our aim. For each paper in the resulting set, coupled with papers found via the citations in search results, we mainly laid our emphasis on hardware and algorithms that are of great importance if we want to construct an intelligent system for elderly care. Through repeated iteration and comparison clustered the technologies illustrated in this literature until we arrive at the taxonomy described in this review.

In summary, in the Introduction, we briefly introduced wearable technologies that should be included to develop smart clothing for elderly people. The rest of this article is organized as follows: Section 2 illustrates indoor positioning technologies. Section 3 presents HAR technologies, mainly sensor-based technologies. In Section 4, we summarized some routinely monitored vital signs and the corresponding methods, such as body temperature, heart rate, etc. At last, in Section 5, we discussed a series of considerations and future trends for the construction of “smart clothing” systems. We believe that our effort will assist researches related to smart clothing platforms or smart home (SH) systems, saving researchers’ time in searching articles and reducing repetitious work.

## 2. Indoor Positioning Systems

Current positioning technologies can be divided into two main categories: outdoor and indoor positioning systems. In outdoor scenarios [10], several well established and widely used navigation systems can provide location services with near meter-level accuracy. These systems include GPS, GLONASS and BDS. However, indoor scenarios constitute 80% of human lives. Indoors, the accuracy of satellite-based positioning decline sharply because of satellite signal losses due to obstructions from buildings, the multipath effect and inconsistent time delay problems. Therefore, these positioning technologies cannot meet the requirements for an indoor location service [19].

Traditionally, indoor positioning systems (IPS) can been considered as systems that function continuously and in real-time to provide the locations of humans or objects in indoor areas [12]. IPSs can be used for many scenarios, e.g., detecting and tracking items, providing assistance for elderly and disabled people in their daily activities, and facilitating medical monitoring for vital signs and emergencies. Some public places such as parks and museums also need indoor positioning services, for example, to provide indoor navigation for blind or visually impaired people, assisting tourists to find their locations to eliminate worries about getting lost, and providing introductory information (or even advertisements) to customers or tourists. Moreover, medical care in hospitals also demands IPSs for tracking patients and expensive equipment, preventing theft, and precise positioning for robotic assistants during surgeries [12,20].

### 2.1. Categorization of IPSs

In recent years, indoor positioning technologies have flourished [9], including both hardware platforms and localization algorithms [12,20]. A variety of sensing technologies have been proposed, such as RFID, WiFi, acoustic signals Bluetooth, and so on [9,10,19,20,21,22,23,24,25,26,27]. These IPSs can be categorized in several different ways according to different criteria. For example, based on system architecture, IPSs can be divided into three classes [9]: (1) self-positioning architectures, where objects determine their locations by themselves; (2) infrastructure positioning architectures, in which the positions of items utilizing the infrastructure are estimated to determine whether items are within the coverage area and to track them; and (3) self-oriented infrastructure-assisted architecture, which depends on a system that computes a position and sends it to a tracked target in response to its request. Alternatively, IPSs can also be categorized according to what they use to determining position. IPSs mainly employ: (1) infrared (IR) technologies; (2) ultra-sound technologies; (3) radio frequency (RF) technologies; (4) magnetic technologies; (5) vision-based technologies; and (6) audible sound technologies [22,23,24]. Other categorizations are possible as well (e.g., based on whether a system requires installation, on sensor types, or on prior knowledge [28,29,30,31,32]).

**Figure 2:**
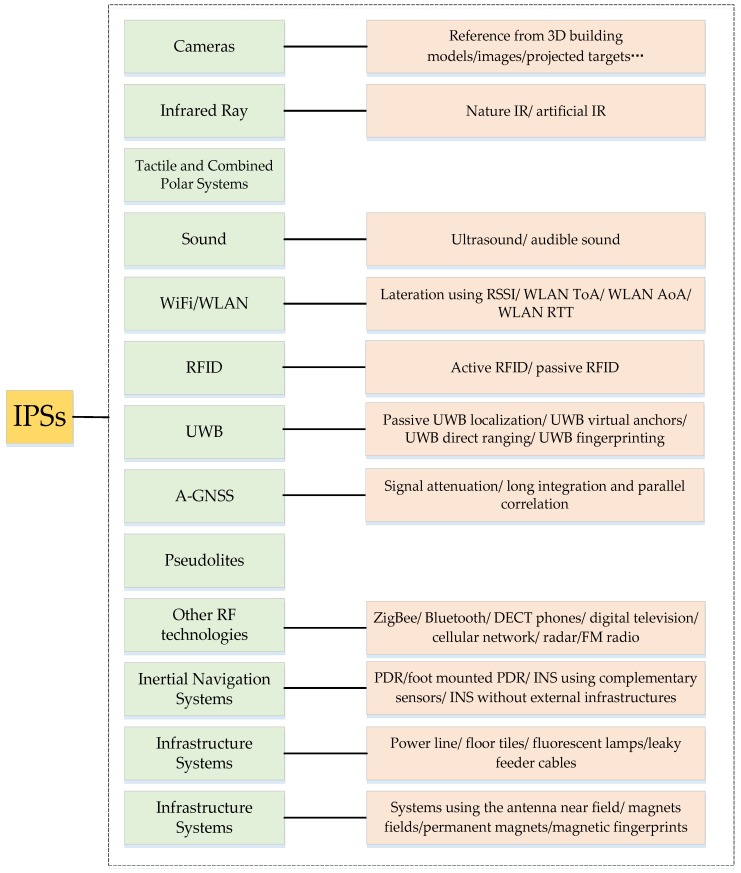
Indoor positioning technologies categorized by Mautz [33].

Among the existing IPS categorizations, this review emphasizes the categorization proposed by Mautz [33], who divided existing IPSs into thirty categories based on sensor type, namely, cameras, infrared, tactile and combined polar systems, sound, WiFi/WLAN, RFID, UWB, Assistant GNSS (A-GNSS), pseudolites, other radio frequencies (e.g., ZigBee, Bluetooth, digital television, cellular network, radar, and FM radio), inertial navigation, magnetic systems, and infrastructure systems, as illustrated in Figure 2.Please refer to [33] for further detail concerning each technology.

### 2.2. Selection of the Proposed IPSs

To select a suitable IPS for elderly care among the existing IPSs, it is essential to develop performance metrics to evaluate the available systems. Typically, accuracy (i.e., position error) is an important performance criterion for IPSs. However, other performance indexes, e.g., cost, availability, privacy and so on, also need to be taken into considerations. Considering that different applications require different types of IPSs—For example, some areas such as supermarkets and private homes pay attention to cost, while others such as medical tracking and indoor navigation systems for vision-impaired people emphasize high accuracy [9]. The following list summarizes some significant performance metrics useful for comparing the various IPSs [24,27,28,29]:

1. *Accuracy*: the average Euclidean distance between an estimated position and the true position [27].
2. *User Privacy*: strict access control to the personal information of individuals [27,33].
3. *Coverage Area*: the area covered by the IPS; this generally includes three levels (i.e., local, scalable, and global [33]).
4. *Required User-Side Device*: whether extra equipment must be carried to construct the IPS.
5. *Cost*: the cost of IPS—this metric mainly includes infrastructure costs (e.g., reuse existing infrastructure or install new devices), installing and maintenance cost (e.g., smartphone, smartwatch, can be reused to decrease infrastructure cost), energy consumption, space occupied, etc. [12,30].
6. *Complexity*: the complexity of designing, constructing, and maintaining an IPS.
7. *Continuity*: the property of continuous operation of an IPS over a contiguous time period to perform its specific function, including acceptable outage frequencies.
8. *Update Rate*: the frequency with which target item positions are calculated (either on devices or at external processing locations).
9. *Data Output*: this metric involves output data types, data output speed, data capture capabilities, data storage, etc.

Table 1 illustrates comparison information for some mainstream and common-used IPS technologies and provides some examples of mature commercial products. 

#### 2.2.1. A-GPS

Assisted GPS (abbreviated as A-GPS or aGPS) can be used to assist positioning where GPS and GLONASS are inadequate because of multipath problems or signal blockage indoors [27,34]. A-GPS can achieve indoor positioning by significantly improving its startup performance—i.e., its time-to-first-fix (TTFF) from a GPS satellite-based positioning system [35]. A-GPS can address the positioning problem resulting from poor satellite signal conditions. However, some standalone GPS navigators used in poor conditions cannot fix their positions because of satellite signal fracture; consequently, they are dependent on better satellite reception levels. Generally, A-GPS systems belong to one of two types: Mobile Station Based (MSB) and Mobile Station Assisted (MSA). Many mobile phones possess this function and often combine with other location services such as WiFi positioning systems or BLE beacon positioning systems.

#### 2.2.2. GSM

Global System for Mobile communication (GSM) or cellular-based positioning systems rely entirely on mobile cellular networks—specifically, on second-generation (or higher) versions of the GSM wireless telephone technology. In most countries, GSM networks are ubiquitous, far outreaching the coverage of WiFi networks. Hence, these wide distributed networks can be used to obtain location estimations for cellphone users. Despite their low accuracy, these positioning technologies attract mass market applications such as pay services and emergency assistance. One of the superior advantages of this technique is that they have little interference because the bands they used are licensed; each licensee avoids interference from other devices operating at same frequency. Besides, GSM also has the merit of 24/7 availability. In the real-world digital human behavior quantification (involves both time and location), systems rely on GSM more frequent than GPS, since GPS, are not reliable (especially indoors) at all time [36].

#### 2.2.3. RFID

RFID technology utilizes radio signals to achieve automatic tracking of people and objects by identifying attached tags containing electronically stored information. An RFID system includes two vital parts: readers and tags. In general, tags are categorized into passive tags and active tags. Passive tags collect energy from a nearby RFID reader’s interrogating radio waves, while active tags have a local power source such as a battery and may be read at distances of hundreds of meters from the RFID reader. In addition, unlike a barcode, the tags do not need to be within the line of sight of the reader; therefore, an RFID tag may be embedded in the tracked object. Readers are preassigned to specially designated places. They communicate using predefined radio frequencies and protocols. Traditionally, RFID technology has been used to detect proximity rather than to determine position [33].

#### 2.2.4. WiFi

WiFi positioning is currently perhaps the most common and widespread location technology. It uses measurements of the intensity of a received WiFi signal (received signal strength indication or RSSI) to achieve a positioning accuracy of between 3 to 30 m [33]. WiFi can reuse the popularity and low cost of existing WiFi networks to construct localization system. In general, existing WiFi positioning techniques can be summarized into four categories as follows: RSSI based, fingerprinting based, Angle of Arrival (AoA) based, and Time of Flight (ToF) based. However, in the majority of instances, WiFi positioning technology is used for proximity detection in public places such as museums, parks, shopping malls, and so on rather than to determine exact positions. Moreover, continuous WiFi scanning for indoor localization will consume a substantial amount of battery power, which makes this technology impractical for long-term use.

#### 2.2.5. UWB

UWB is a radio technology that can use very low energy signals for short-range, high-bandwidth communications over a large portion of the radio spectrum. UWB can be used for precision location and tracking applications by detecting the time difference of arrival (TDOA) of RF signals to calculate the distance between a reference point and the target [33]. Many applications already use UWB positioning techniques, such as real-time indoor precision tracking for mobile inventory, locator beacons for emergency services and indoor navigation for blind or visually impaired people. Hence, UWB is one of the most accurate and promising technologies to realize accurate indoor positioning despite its high costs.

#### 2.2.6. DR

DR uses a previously determined position and tracks changes to infer the current position. It follows the current position based on both past known and estimated velocities over time and heading direction [44]. DR systems are most often constructed using Inertial Measurement Unit (IMU) devices that contain accelerometers used for step detection and step length estimation and magnetic compasses or low-cost gyros for heading determination [45]. If an initial location is known, based on continuous updates of the travelled distance and heading, the current position can be propagated without the need to acquire an external reference position. Although DR can provide reliable and always-available position information, it suffers from significant errors due to imprecise speed and direction estimates.

#### 2.2.7. Infrared

Infrared positioning technology utilizes both natural and artificial light whose spectrum differs from visible light and terahertz radiation. Consequently, this technology is unobtrusive for humans compared with indoor positioning technologies based on visible light [33]. Typical infrared positioning systems can be divided into two types: direct infrared systems and diffuse infrared systems. A direct infrared system uses a point-to-point ad-hoc data transmission standard to achieve very low-power communications, while diffuse infrared systems use wide angle LEDs to emit signals in many directions. There are three IPS approaches that use infrared technology: Proximity, differential phase-shift, and angle of arrival (AoA).

#### 2.2.8. BLE Beacon

Bluetooth Low Energy (BLE) technology achieves peer-to-peer communications while consuming only small amounts of power. Based on BLE beacons, an energy-efficient system can be constructed for indoor positioning. Similar to WiFi, Bluetooth modules are already included in most commercial mobile devices; therefore, no extra device is required on the user side. Because Bluetooth beacons broadcast their unique identifiers to nearby portable mobile devices and can trigger a location-based action on these devices, no paired connection with the mobile device is needed [46]. Thus, BLE beacon based IPS is a competitive technology because it can achieve an acceptable localization accuracy and consumes less energy than GPS and WiFi approaches.

#### 2.2.9. Acoustic Signal

Acoustic signal systems use echolocation to form a location estimate. A pulse outside the human audible range is emitted by a speaker tag attached to the user and received by a series of microphones installed in ceilings [47]. The distance of the speaker tag from the microphone can then be estimated by measuring the speed of the traveling sound waves, while multiple receivers allow angles to be determined. Acoustic signal systems are effective for room level accuracy, but suffer from sound reflections, which limits their absolute accuracy. Moreover, large numbers of receivers are required to achieve centimeter-level accuracy for a given coverage area, thus increasing the system’s cost.

#### 2.2.10. Visible Light

Visible Light Positioning (VLP) is an emerging positioning technique based on Visible Light Communication (VLC), which uses light emitted by diodes (LEDs) to transmit digital information [28]. The information delivered by these light signals can be used to determine the position of a person or object within a room. Buildings already have a large number of light fixtures that cover the entire structure, so these fixtures potentially represent a large number of transmitter locations, allowing a much higher transmitter density than other technologies. Because light does not travel through opaque boundaries such as walls or floors, the signal is localized to the room in which it is transmitted. This also eliminates interference between transmitters in adjacent rooms, allowing high spatial reuse of bandwidth. Moreover, light based positioning raises fewer privacy concerns because covering the receiver can guarantee the system is not in use.

#### 2.2.11. Image-Based IPS

This approach uses optical information to realize indoor positioning; therefore, it is also known as optical indoor positioning [33]. In this system, a camera such as a mobile phone camera, an omnidirectional camera or a three-dimensional camera is used as the only or main sensor. The acquired images are combined with computer vision technologies to achieve indoor positioning. In general, this technology is easily affected by environmental factors and requires significant amounts of image processing computation. In addition, providing coverage over multiple rooms requires a prohibitively expensive number of cameras, and positioning performance suffers unless known reference markers are attached to the objects being tracked.

#### 2.2.12. Geomagnetism-Based IPS

Modern buildings with reinforced concrete and steel structures have unique, spatially varying ambient magnetic fields that can be used for positioning, in much the same way (albeit on a much smaller spatial scale) as animals use the Earth's magnetic field [48]. In principle, a non-uniform ambient magnetic field produces different magnetic observations depending on the path taken through it. Therefore, indoor positioning can be achieved by using the anomalies (fluctuations) in these ambient magnetic fields. This approach has been facilitated by modern smartphones and the rapid development of sensor technology. Generally, geomagnetism-based indoor positioning needs only a three-axis magnetometer such as a smartphone’s compass to precisely locate individuals within indoor spaces. These systems can achieve positioning errors below 6 feet because each building or structure has a unique magnetic “fingerprint”.

For elderly care scenarios, the two-dimensional (2D) accuracy should be between 0.5 m and 1 m, and the update rate cannot be slower than 0.5 s. Moreover, the crucial criterion “user acceptance” should be taken into consideration. The user acceptance criterion defines how intrusive a system is to the user—for example, whether the elderly need to remember to accommodate the system by wearing tags [33]. Table 2 lists specific criteria and brief descriptions for elderly care systems [33,37,41].

## 3. Human Activity Recognition

**Figure 3:**
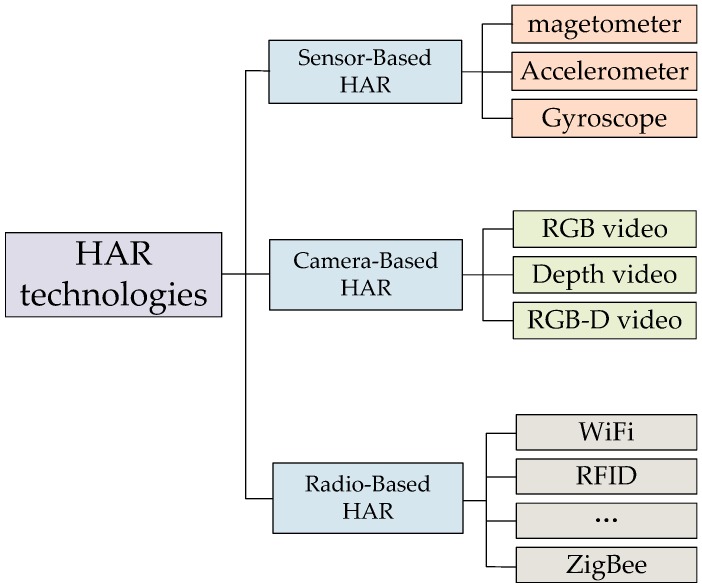
Categorizations of HAR systems.

HAR during daily life is another fundamental function for elderly care system because HAR can provide assistance services. Continuous monitoring of elderly activities allows the detection of abnormal situations and can help ameliorate the effects of unpredictable events such as sudden falls. These capabilities are required for this type of wearable system to assist the elderly in their daily lives and increase their safety. As illustrated in Figure 3, the current wearable technologies that can be used to implement HAR can be summarized into three categories: (1) Vision-based recognition use cameras to record video sequences and recognize activities by combining the images with computer vision algorithms. In camera types used include RGB video, depth video and RGB-D video [49,50,51,52,53]; (2) Radio-based recognition systems use technologies, such as ZigBee, WiFi, RFID, etc., to infer human activities from the status of utilized objects or from changes in environmental variables [54]; (3) Sensor-based recognition systems employ on-body (wearable) sensors such as accelerometers and gyroscopes to detect the movements of body parts [13].

The vision-based systems are easily influenced by lighting variability and other exogenous factors; consequently, their recognition accuracy decreases from laboratory environments to outdoor environments due to inevitable visual disturbances [14]. In addition, regardless of how many 2D/3D cameras are employed and installed (e.g., a defined number and type of cameras in specified areas) continuous monitoring is still restricted to the camera locations. Because of these limitations, vision-based HAR systems are not well suited to most elderly care applications. In term of radio-based HAR system, the base stations must be prearranged and the tags are often attached to a person's wrist, ankle, head, or other parts. By observing that different human activities cause different wireless communication patterns between the attached tags and the base station, human activities can be recognized. However, these technologies also suffer from a similar drawback as the vision-based technologies: radio-based HAR does not work in areas where a base station is unavailable [55]. Consequently, radio-based HAR systems are also not a suitable scheme for most elderly care situations.

Luckily, with the rapid development of Micro-electromechanical Systems (MEMS) sensor technologies such as gyroscope, acceleration and magnetic sensors, sensor-based HAR technologies, particularly wearable sensor-based HAR, are becoming more and more suitable for use in ambient assisted living (AAL) systems or platforms, particularly for applications involving remote and continuous monitoring of elderly people [56]. Sensor-based HAR systems have several advantages. First, because of advances in MEMS technologies and their widespread use, these sensor devices have become cheaper. Moreover, they have become small and lightweight enough to carry. In addition, sensor-based systems do not need base stations such as cameras pre-installed in specific locations; therefore, it is possible to use them to achieve continuous monitoring beyond carefully restricted environments. Moreover, these systems collect data for activity recognition in a passive way; thus, they do not create electromagnetic pollution that exposes people to potentially negative health effects. Finally, sensor-based systems consume only small amounts of power when collecting data for HAR because of rapid advances in the techniques used to manufacture them [51,56,57].

**Figure 4:**
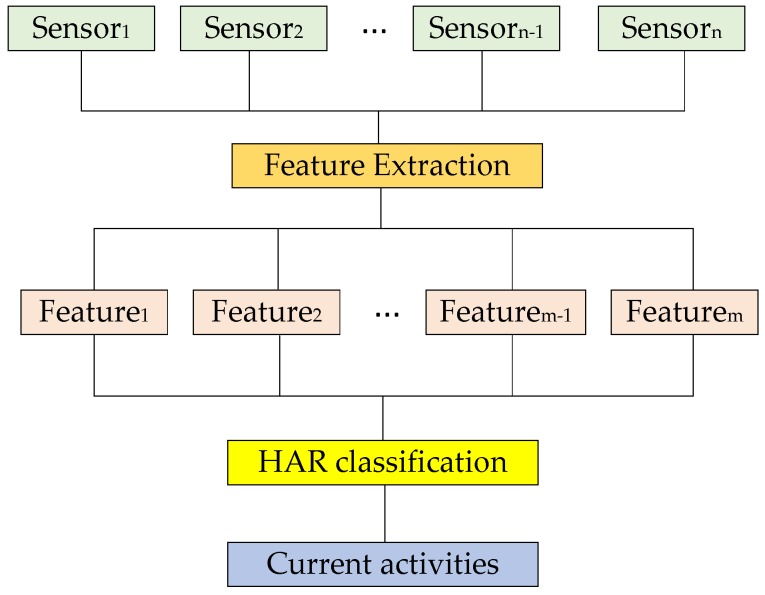
Typical flowchart of a HAR system.

Among these sensors, accelerometers have been given the most attention in HAR. For example, Mannini et al. [58] and Pirttikangas et al. [59] used accelerometers to identify both normal and abnormal activities such as lying down, walking, standing, reacting to chest pain, and so forth. However, other sensors such as gyroscopes, magnetometers, and barometric pressure sensors have been combined with accelerometers to improve activity recognition performance [60]. In [61,62,63,64], researchers combined accelerometers with gyroscopes to recognize fall detection, gait analysis, and gesture recognition activities. In general, depending on the type of activity being recognized, the body position, the classification method and the feature set being used, both accelerometers and gyroscopes are capable of taking the lead role in the activity recognition data-acquisition process. For example, walking upstairs and downstairs are activities better recognized from gyroscopic data in most situations. In contrast, activities such as standing and sitting are better recognized from accelerometer data. For walking, biking and jogging activities accelerometer data performs slightly better than gyroscope data. However, in most circumstances, the accelerometer acts as the lead sensor while the gyroscope functions as a supplementary sensor. Figure 4 illustrates a typical flowchart for a behavior recognition system. Features are extracted from several different sensor sources and input into behavior classification algorithms to be classified as a specific activity.

### 3.1. Sensor Placement

The sensor placement of wearable devices refers to the locations where the sensors are placed and how the sensors are attached to those locations. For elderly healthcare, we need to not only monitor normal activities such as standing, sitting, walking, biking, jogging, lying, and climbing upstairs and downstairs but also recognize abnormal activities such as forward falls, backward falls, chest pains, fainting, vomiting, and headache [65,66]. Emphasizing sensor placing and sensor type selection is important because wearable sensor placement has a direct effect on the recognition accuracy of body motions [67] and because different sensors (e.g., gyroscopes or accelerometers) are respective important in different situations. For example, if the wearable sensors are placed around the waist, the gyroscope data is better for recognizing stair climbing and descending activities in most situations, whereas standing and sitting activities are better recognized by the accelerometer. For walking, biking and jogging activities, the accelerometer data is slightly better than the gyroscope data [66].

**Figure 5:**
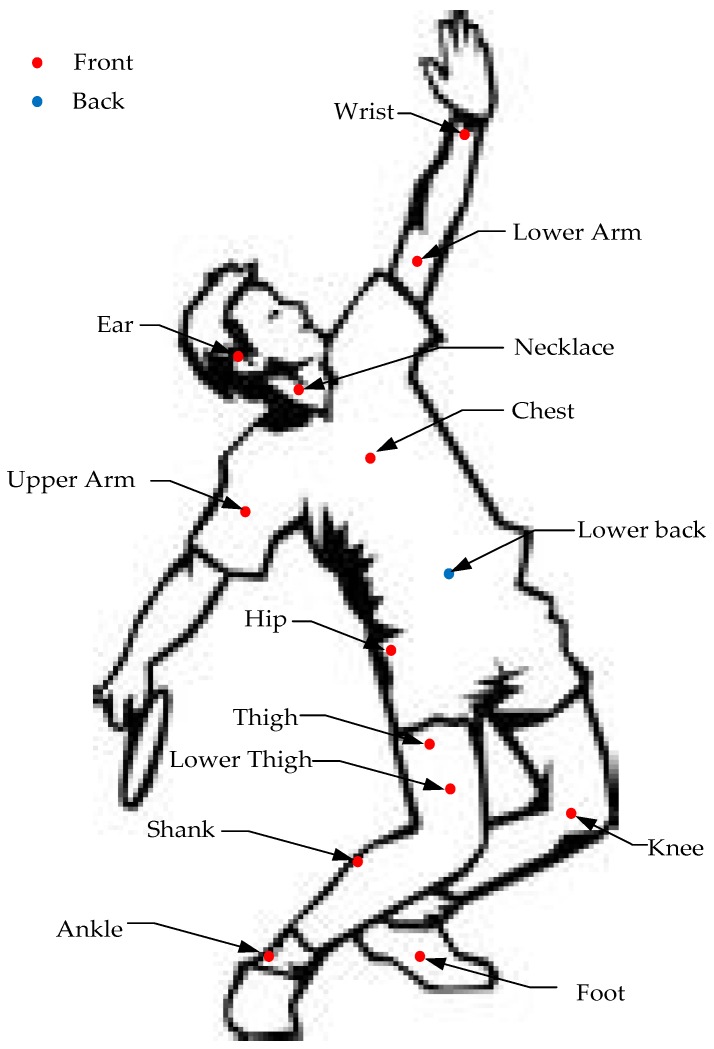
Graphical demonstration of sensor placement.

Figure 5 illustrates common locations for sensor placement according to the majority of past studies. Table 3 summarizes the past studies on sensor placement for HAR. The bulk of these studies report that recognizing more complex activities requires multiple sensors placed in various locations. Most existing work uses the accelerometer as the lead sensor, while the gyroscope is used as a supplementary sensor that can improve the recognition performance—including both the number of recognizable activities and the recognition accuracy [58,59,68,69,70,71,72]. If merely recognizing normal activities, an accelerometer located on the wrist can meet this demand [58]. In addition, researchers also prefer to place sensors for normal activity recognitions on the sternum, the lower back or foot. Indeed, waist-placement of wearable sensors can better represent most human motions because they are then close to the center of mass of the human body [73]. Besides, sensors or devices can be easily attached to or detached from a belt worn at waist level. Therefore, waist-placement causes less constraint in body movement and minimizes discomfort. A range of basic daily activities, including walking, postures and activity transitions have been classified in previous studies according to the accelerations measured from a waist-worn accelerometer [13,58,71]. Moreover, foot-attached sensors can significantly reflect gait-related features during locomotion or walking. Steps, travel distance, velocity, and energy expenditure can be estimated by a foot-worn accelerometer [58,68,69,74] Sensors can also be located around the thigh, the majority of cellphone based HAR studies investigated putting the embedded sensors into pockets; their results showed that thigh-located sensors obtained high recognition performance for the leg-involved activities which many people perform regularly in their daily routines, i.e., walking, jogging, riding, running, ascending, descending, etc. [59,60,68,74,75,76].

The combination of accelerometer and gyroscope may improve the overall recognition performance—or at least keeps it equal to the maximum of their individual recognition performances regardless of sensor locations with very few exceptions. According to Shoaib et al. [66], who studied the performance of accelerometer and gyroscope in detail with respect to different sensor locations, both individually and in combination. Body locations such as on the wrist, chest, hip, thigh, and upper arm have been used to place accelerometer or gyroscope to identify activities such as lying down, sitting, walking, running, cycling, climbing stairs, descending stairs, and jogging. Based on their evaluation and analysis, the recognition performance of accelerometer or gyroscope depends on the sensor placement, but the overall performance is enhanced in combination. Other researchers have also investigated the optimal placement of accelerometers for human activity recognition. Chamroukhi et al. [69] evaluated the influence of the sensor configurations and their placements on the precision of HAR. The best results were obtained for a configuration with three sensors placed on the chest, thigh and ankle. These results showed that HAR could be significantly improved by combining accelerometers located on both the upper and lower part of the human body.

How to place sensors on human body is also a research- worthy problem. The majority of researchers chose indirect forms of attachments such as straps, belts, wristbands, or other accessories to prevent relative motion between the sensors and the parts of the human body [59,60,61,62,63,66,67,68,69,70,71,72,73]. Moreover, sensors and wearable devices can also be directly placed into pockets or attached to other parts of clothing; this was especially prevalent in the smartphone-based HAR studies. However, these pocket-placed sensors must be guarded against movement, otherwise vibration and displacement can affect the wearable systems and decrease recognition accuracy.

### 3.2. Features for Classification

HAR can also be regarded as a pattern recognition problem [66]. To extract features from raw data in real time, sliding window techniques are generally used. These sliding windows divide continuous signals into small time segments. Then segmentation and classification algorithms are employed on the data in each window. In the feature extraction step, signal characteristics such as time-domain and frequency-domain features are widely used for raw data pre-processing, as illustrated in Table 4. Time-domain features include mean, variance, median, skewness, kurtosis, and so on, while frequency domain features generally include peak frequency, peak power, spectral power at different frequency bands and spectral entropy.

In general, feature selection is a process to collect relevant information and obtain quantitative measures that allow patterns to be compared. The accuracy of activity recognition is dependent upon feature selection. Usually, a single feature is insufficient to satisfy the demands of recognition of several activities. Consequently feature sets that combine several relevant features are used for complex activity recognition [66]. Compared with frequency-domain features, time-domain features require less computation and storage; however, Fourier transformations have a high computational cost. Despite this cost, some studies still choose feature sets containing frequency domain features to improve the activity recognition results. For example, Shoaib et al. [66] used a combination of spectral energy, mean, variance, and other features to demonstrate the roles of an accelerometer and a gyroscope in a HAR system, the results shows that frequency-domain techniques perform well in capturing the repetitive nature of sensor signals. This repetition often correlates to the periodic nature of a specific activity such as walking or running.

Moreover, to extract the most relevant features to construct feature sets that can reduce computational requirements and simplify the recognition models, many researchers use principal component analysis (PCA), Linear Discriminant Analysis (LDA), Independent component analysis (ICA), Factors Analysis (FA) and Minimum Description Length (MDL) to reduce redundant or irrelevant features in feature sets that can negatively affect the recognition accuracy [78,79,80,81]. The Minimum Redundancy and Maximum Relevance (MRMR) method is also utilized in feature set construction [82]. In that work, the minimum mutual information between features is used as a criterion for minimum redundancy and the maximal mutual information between the classes and features. Similarly, Maurer et al. [83] applied a Correlation-based Feature Selection (CFS) approach for the evaluation of feature set, taking advantage of the fact that this method is built into WEKA [84]. CFS works under the assumption that features should be highly correlated with a given class but uncorrelated with each other. Readers should refer to [66] for detailed information concerning how to assess a feature set.

### 3.3. Algorithms for Sensor-Based HAR

In terms of the classification techniques used for HAR, current studies can be categorized into three types: data-driven, knowledge-driven and hybrid approaches. The data-driven approaches first collect sensor data and then exploit the unseen correlations between activities and sensor data. Finally, they establish a model to classify the activities. The model focuses on the use of probabilistic and statistical analysis methods and is suitable for single-user single-activity scenarios. Some commonly used data-driven approaches are the Hidden Markov Model (HMM) [57], *k*-Nearest Neighbor algorithm (KNN) [13,60,68,74,85,86], Principal Component Analysis (PCA) [87], Naïve Bayes (NB) [13,59,60,74,86], Gaussian Mixture Model (GMM) [58], Random Forest (RF) [85], Decision Tree [13,59,74,86], and so on. In contrast, knowledge-driven approaches start with an abstract model representing common knowledge and then train and assess the model using sensor data. In this algorithm, the models are established from the mappings from inputs (data) to outputs (activity labels). These approaches utilize machine learning techniques to extract human activity patterns from daily activities and, subsequently, use the learned patterns as predictive models. Such algorithms include neural networks and linear or non-linear discriminant learning [76], expressive description logics (DLs) [88], possibly coupled with rule-based languages [89,90], etc.

However, both data-based and knowledge-driven approaches have limitations. For instance, data-based techniques suffer from shortcomings when applied to the recognition of complex high-level activities, especially in terms of portability, extensibility, and support for common-sense knowledge, whereas knowledge-based systems carry other restrictions such as lacking support for probabilistic reasoning, making them unable to handle the variability of complex activity execution in combination with the inherent uncertainty of sensed and inferred context data [57]. Hybrid methods are combinations of data-driven and knowledge-driven approaches. These methods aim to cope with the limitations of both data-driven and knowledge-driven techniques. For example, Riboni et al. [89,90] proposed using ontologies and ontological reasoning combined with statistical inference methods for context-aware activity recognition. This approach can solve the scalability problem supervised learning algorithms when the number of considered activities and the amount of contextual data are large. Lester et al. [91] studied a hybrid approach that combines boosting to discriminatively select useful features and train an ensemble of static classifiers to recognize different activities with HMMs to capture the temporal regularities and smoothness of activities to classify human activities.

Table 5 presents summaries of human activity classification systems. In the following, we briefly introduce the commonly used classification algorithm, i.e., HMM, KNN, SVM, RF, GMM, NB, and so forth.

#### 3.3.1. Hidden Markov Models

HMMs are statistical Markov models in which the system being modeled is supposed to be a Markov process with unobserved (hidden) states. An HMM can be taken as the simplest model of a dynamic Bayesian network. Generally, in simple Markov models (such as a Markov chain), the state (activity) is directly visible to the observer; therefore, the state transition probabilities are the only parameters. However, in a HMM, the state is not directly visible; only the output, which is dependent on the state, is visible. Each state has a probability distribution over the possible output tokens. Therefore, the sequence of tokens generated by an HMM provide some information about the sequence of states. The adjective “hidden” refers to the state sequence through which the model passes, not to the parameters of the model; the model is still referred to as a “hidden” Markov model even when the parameters are known exactly.

Lester et al. [91] used an HMM as one part of a hybrid classification system to capture the temporal dynamics between numerous daily activities, rather than directly using raw features. The HMMs were trained via the posterior probabilities of the decision stump in order to use the results from discriminatively trained classifiers, as well as to reduce the complexity of the HMMs. Attal et al. [56] took advantage of the statistical models used in the HMMs, including both the sequential aspect and the temporal evolution of the data, to improve the classification performance for daily physical activities under unsupervised learning.

#### 3.3.2. *k*-Nearest Neighbor

KNNs are a memory-based model defined by a set of objects known as examples (a training set) for which the outcome is known (i.e., the activities are labeled). Each example consists of a data case with a set of independent values labeled by a set of dependent outcomes. The independent and dependent variables are categorical when the task is classification. Given a new observation, the KNNs algorithm can estimate the outcome based on training set. KNN achieves this by finding *k* examples that are closest in distance (such as Euclidean distance) to the *k* neighbors (hence, the name *k*-Nearest Neighbors). Generally, the choice of the *k* value is critical in building the KNN model. In fact, *k* can be regarded as one of the most important factors of the model because it can strongly influence the quality of predictions [56]. The KNN algorithm has a nonparametric architecture and its advantages are that it is simple, straightforward, flexible to implement and requires no training time. However, it suffers from drawbacks including intensive memory requirements and slow estimation.

Moncada et al. [68] used the KNN classifier to discriminate among 16 daily activities of six healthy subjects under laboratory conditions using inertial devices enhanced with barometric pressure sensors to capture activity-related data. They achieved overall classification accuracy rates of nearly 93% and more than 95% for the repeated holdout and user-specific validation methods, respectively, for all 16 activities. Based on data from the UCI HAR and UCI PAMAP2 datasets, Morillo et al. [96] compared the KNN algorithm with other algorithms to study how the selected classification method affected energy consumption on a smartphone when running a HAR algorithm. The results show that KNN requires more memory and consumes more energy than the C4.5, SVM, NB, and other methods.

#### 3.3.3. Support Vector Machines

SVMs are data-based machine learning models with associated learning algorithms that analyze data used for classification analysis. Given a set of labelled training examples belonging to one of two categories, an SVM training algorithm builds a model that subsequently assigns new examples into one category or the other, making it a non-probabilistic binary linear classifier. An SVM model represents the examples as points in space, mapped so that the examples belonging to each category are divided by a clear gap that is as wide as possible. New examples are then mapped into that same space and predicted to belong to a category based on which side of the gap they fall on. However, because example data are often not linearly separate, SVM introduced the perception of a “kernel induced feature space” which casts the data into a higher dimensional space where the data are separate. Overall, SVM is intuitive, theoretically well-founded, and has been shown to be successful in practice.

Wang et al. [97] performed a comparison of the KNN and SVM classifiers by employing self-defined features, also known as ensemble empirical mode decomposition (EEMD)-based features. Different accuracies were obtained by the two classifiers which work differently to classify the activity data for “standing up after lying down” acquired from sensors on the left ankle. Atallah et al. [85] combined a generative model (multiple eigenspaces) with SVM training on partially labeled training data into a single activity recognition framework intended to reduce the amount of supervision required and improve the recognition accuracy. To reduce the computational energy consumption, a modified SVM algorithm was proposed by Anguita et al. in [93], namely, the novel Hardware-Friendly SVM (HF-SVM) for multiclass activity classification. This technique employed the standard SVM and exploited fixed-point arithmetic to reduce the computational cost.

#### 3.3.4. Random Forests

RFs are an ensemble learning technique for classification that operate by constructing a multitude of decision trees at training time and, finally, outputting the class representing the mode of the classes (classifications) of the individual trees. The algorithm begins by selecting many bootstrap samples from the data. Typically, in these samples, about 63% of the original observations happen a minimum of one time. When the observations from the original set do not occur in a bootstrap sample, they will be called “out-of-bag” observations. A classification tree is applied to each bootstrap sample, however each node can employ only a limited number of randomly selected variables (e.g., the square root of the number of variables) for binary partitioning. The trees are fully grown and then each is utilized to predict out-of-bag observations. The predicted class of an observation is computed by majority vote of the out-of-bag predictions for that observation; ties are split randomly [98].

Bedogni et al. [95] presented a classification methodology to identify a user’s activity, i.e., driving a car, riding in a train, or walking, by comparing different machine learning techniques (RF, SVM and NB). The recognition results demonstrated that the RF algorithm provide the highest average accuracy, outperforming the SVM and NB.

#### 3.3.5. Naive Bayes

Naive Bayes classifiers are a set of simple probabilistic classifiers based on the application of Bayes’ theorem with a strong (naive) independence hypothesis between every pair of features. This method appears to work well in practice even when the assumption of independence is invalid. In general, NB classifiers are highly scalable, but they require a number of parameters that vary linearly according to the number of features (predictors) in a learning problem. Maximum-probability training can be employed by assessing a closed-form expression, which takes linear time, instead of the computationally expensive iterative approximation algorithms that are used in many other types of classifiers. The simple structures of NB methods give them several advantages: they are easy to construct, their inferences (classifications) can be obtained in linear time (in contrast, the process of inference in Bayes networks with a general structure is known to be NP-complete), and finally, because the process of constructing an NB is incremental, it can be easily updated; in other words, this algorithm can quickly be adapted to consider new cases [59,74,86].

Mannini et al. [58] compared the performance of single-frame classifiers such as SVM, KNN, GMM, and so forth in HAR based on smartphone sensor data. The results show that the NB classifier performed better than GMM and an artificial neural network in this scenario. To explore the individual or combinatorial recognition performance of accelerometer and gyroscope data for different activities, Shoaib et al. [66] used NB to analyze the role of an accelerometer or a gyroscope.

#### 3.3.6. Gaussian Mixture Models

A GMM is a probabilistic approach that uses a weighted sum of Gaussian component densities. Often, data are partially Gaussian distributed, in which case the data set can be supposed to have been made by a hypothetical Gaussian mixture. Due to its probabilistic nature, a GMM is generally preferred over models that separate a data set into discrete parts. GMM parameters (i.e., the proportions, the mean vectors and the covariance matrices of the Gaussian components) are estimated from training data using the iterative Expectation-Maximization (EM) algorithm or through Maximum a posteriori (MAP) estimation from a well-trained prior model [99].

Mannini et al. [58] demonstrated that GMMs can be an important tool for classifying HAR data; however, the classification accuracy they obtained from a GMM was worse than the accuracy achieved by these authors’ self-defined cHMM-based sequential classifier in that scenario. Srivastava et al. [100] proposed using GMMs to construct a hierarchical human activity recognition system to detect continuous human daily activities. The recognition accuracy ranged from 70.4%–98.8%.

#### 3.3.7. Decision Tree

A decision tree is a schematic, tree-shaped diagram used to map observations about an item to reach conclusions about the item’s target value or to show a statistical probability. Each branch of the decision tree visually and explicitly represents a possible decision in the decision making process. In general, when the trees model a process in which the target variable can take a finite set of values, they are called classification trees. These classification tree structures illustrate how and why one choice may lead to the next; the branches indicate that each option is mutually exclusive. Hence, decision tree methods allow users to depict a complex problem in a simple, easy-to-understand format that shows the relationship between different decisions.

The most commonly used decision classifier is the “J48” classifier, which is available in the WEKA toolkit. Khan et al. [94] trained this classifier from a dataset containing four human activities (lying down, sitting, walking and running). The J48 classifier correctly classified these activities 92.74% to 98.9% of the time (average: 96.61%, standard deviation: 1.63). Bonomi et al. [72] classified 20 activity types classified using a decision tree algorithm from data collected using only one tri-axial accelerometer mounted on the lower back.

#### 3.3.8. Artificial Neural Network

Artificial Neural Networks (ANNs) are a form of artificial intelligence that attempts to mimic the behavior of the human brain and nervous system. Typically, the structure of ANNs consist of a number of processing elements (PEs), called nodes, which are usually arranged in various layers: an input layer, an output layer and one or more hidden layers. The propagation of information in ANN begins by presenting the input data to the input layer. The network regulates its weights according to a training dataset and uses a learning rule to allocate a set of weights to nodes to create the input/output mapping that has the smallest possible error. This process is called “learning” or “training”. After the training phase of the model has been successfully completed, the trained model must be validated using an independent testing set. This modelling philosophy is similar to the conventional statistical models in the sense that both approaches are trying to establish the relationships between a historical set of model inputs and corresponding outputs.

Ronao et al. [76] assessed the suitability of a convolutional ANNs to identify human activities using time-series accelerometer and gyroscope sensor data from a smartphone. They found that the ANNs outperformed all other state-of-the-art algorithms at HAR, including the NB, SVM, and Decision tree algorithms. The ANNs particularly outperformed SVM, presenting noticeably better performance in classifying moving activities.

#### 3.3.9. Deep Learning

Deep Learning (DL) is a new area of Machine Learning research. Compared with ANNs, DL makes Machine Learning much closer to one of its original goals: Artificial Intelligence. Actually, the concept of deep learning is developed from the study of ANNs. Its motivation is also to establish and simulate the learning mechanism of human brain to interpret the data. Thus, deep learning and ANNs own many similar characteristics such as the hierarchical learning structure. More generally, DL is an ANN with multiple hidden layers of units between the input and output layers. Through combining low-level features to form a more abstract high-level representation of attribute categories or features, DL can be used to find the distributed feature representation of data. To realize this goal, the training mechanism of DL is changed from back propagation (used by ANNs) to layer-wise strategy. Restricted Boltzmann machine (RBM) are DL algorithms commonly used today. A restricted Boltzmann machine (RBM) is a special type of Random Neural Network (RNN) model that has a two- layer architecture, symmetric connections and no self-feedback. The two layers in an RBM are fully connected whereas there are no connections within the same layer. This algorithm has many advantages. For instance, an RBM provides an effective method for detecting features. When a feedforward neural network is initialized with an RBM, its generalization capability can be dramatically improved. A deep belief network made up of several RBMs can detect more abstract features than other methods. Due to these advantages, RBMs have wide applications in deep learning [101].

Bhattacharya et al. [92] investigated RBM as applied to HAR. Contrary to expectations, their results indicate that using RBM models for HAR can result in acceptable levels of resource use even for low-power hardware devices such as smartwatches that are already on the market. Alsheikh et al. [102] used a mere triaxial accelerometer and RBMs for HAR. In their model, the spectrogram signals from accelerometer were continuously fed to a deep activity recognition model. Therefore, the pre-layer of this model was chosen as a Gaussian-binary RBM (GRBM), which can model the energy content in the ongoing accelerometer data. Then, the subsequent layers were binary-binary RBMs (BR BMs). According to their results, when the window size was 10 s, the accuracy of DL models reached to 98.23%, which was better than LR, C4.5, and multilayer perceptron (MLP).

#### 3.3.10. Other Algorithms

C4.5 is a statistical classifier that derives from the Iterative Dichotomies 3 (ID3) and is used to build a decision tree from a set of training data. This algorithm introduces an alternative formalism including a list of rules of the form “if A and B and C and ... then class X”, where the rules for each decision are grouped together. A decision is made by finding the first rule whose conditions are satisfied by the decision; if no rule is satisfied, the decision is assigned to a default class. Compared with other classification algorithms, C4.5 is both computationally efficient and lightweight and is suitable for implementation in resource-constrained devices [70]. This algorithm is used in [58,60,76,83,96] for HAR classification purposes. Refer to [83], C4.5 was chosen as the classifier because it provides a good balance between accuracy and computational complexity.

Logistic regression (LR) is a regression classification model with a binary nature. It hypothesizes that the data values are precisely pre-determined, but this is not accurate for all conditions. Some uncertainty data appears in many applications because data collection methodologies from physical experiments often result in repeated measures, outdated sources and imprecise measurements. The LR model learns this uncertainty data by estimating probabilities using a logistic function—the cumulative logistic distribution. The LR algorithm is also built-in to WEKA. Kwapisz et al. [75] used LR to identify normal human activities; the results showed that LR outperformed the C4.5 and MLP classifiers when classifying walking activities.

A MLP is a feedforward ANN model that maps sets of input data onto a set of appropriate outputs. Conventionally, MLP consists of three or more layer of nodes in an oriented graph in which each layer is fully linked to the next one in the network. Nodes in the input layer represent the input data. All the other nodes map inputs to outputs using a linear combination of the input with the node’s weights and biases and by applying an activation function. MLP can be used to classify data that are not linearly separable. Kwapisz et al. [75], using features such as average, standard deviation, average absolute difference, and so on, compared the accuracy performance of algorithms such as C4.5, LR, and MLP and reported that MLP outperformed the other classifiers on HAR data collected from a thigh-worn accelerometer.

## 4. Vital Sign Monitoring

As people grow older, most elderly people suffer from some age-related problems such as hypertension, diabetes, coronary disease, hyperlipoidemia, and so forth. Therefore, it becomes essential to design a continuous and real-time health monitoring and evaluation system to ensure the elderly can have healthy daily lives. Fortunately, due to advances in sensor technology, low-power microelectronics and wireless communication standards gerontechnologies are becoming increasingly commonplace in our society. In particular, advances in wearable and non-invasive sensors make it possible to regularly, comfortably and continuously monitor human vital signs for improved in-home healthcare. Regular vital sign monitoring is crucial in constructing the health baseline for an individual and alerting users and medical professionals of risky situations when further medical attention and care may be necessary.

Here, we limit the discussion to wearable and non-invasive biosensors, omitting implantable devices. Such sensors can be worn in contact with or near to the body and can measure an impressive variety of vital signs. Four main vital signs are routinely monitored by medical professionals: body temperature, heart rate, respiration rate, and blood pressure [103]. Therefore, we first summarize the techniques to detect and monitor these bio-signals. In addition, other bio-signals such as pulse oxygenation (oxygenation of fresh arterial blood) and blood glucose are also widely used by medical professionals, although they do not yet fall into the category of main vital signs. However, we also present some wearable technologies that can monitor these vital signs. Table 6 briefly summarizes some human vital signs that have been successfully monitored using wearable sensors in past studies.

### 4.1. Body Temperature

A person’s body temperature (BT) is an important vital sign that can provide an insight into their physiological state. The normal core body temperature of a healthy, resting adult is stabilized at approximately 37 degrees Celsius. This temperature fluctuates due to changes in the metabolism rate. For example, body temperature is relatively lower in the morning because a resting body has a slower metabolic rate, and it is higher at night, after daytime muscular activity and food intake. In general, an abnormal body temperature is an indicator that a person may be suffering from an infection, fever or low blood flow due to circulatory shock. When measuring body temperature, the choice of measurement site is important because body temperature varies when measured at different locations. For instance, normal oral temperature (usually considered as the standard measurement for normal core body temperature), is approximately 37 degrees Celsius, whereas a rectal temperature (which is the most accurate type of body temperature measurement) is typically fall approximately 37.6 degrees Celsius when taken at room temperature [103,118].

Body temperature can be monitored by using thermistors, the thermoelectric effect, or by optical means [103]; however, the most commonly used technique for non-invasive and wearable temperature measurement is the thermistor. Using a negative temperature coefficient (NTC) resistor as a temperature sensing element, Chen et al. [105] proposed and demonstrated a design for a non-invasive temperature monitoring scheme in which conductive textile wires are used to integrate the sensors into a wearable monitoring platform such as a baby's smart jacket. Similarly, a textile-based temperature sensor was manufactured on an industrial-scale flat-bed knitting machine by incorporating the sensing element into a double layer knitted structure [104]. Nickel and tungsten wires proved to be good candidates for the sensing elements in temperature sensitive fabric due to their high reference resistance, sensitivity and availability. The resulting sensing fabric can be applied to make wearable skin temperature measurements from the wearer. Moreover, many commercially available thermistor and temperature ICs already exist, such as LM35. These can be attached directly to the skin; however, note that skin temperature measurements by wearable sensors may not reflect the body's core temperature, so a calibration algorithm is needed to establish the relationship between the two temperature measurements [106].

### 4.2. Heart Rate

Heart rate (HR) or pulse is unarguably the most pivotal variable in a human body. The heart must be in perfect working condition for a person to be considered healthy. The human heart is primarily in charge of pumping oxygenated blood and nutrients to all the parts of the body and through the organs that remove carbon dioxide and other wastes. Generally, any major changes in a person’s physical or mental state usually result in pulse changes. The HR of a healthy resting adult ranges from 60–100 beats per minute. However, the HR frequency of any individual varies from this baseline depending on their activity and physiological state. For example, during sleep, a slow heartbeat of approximately 40–50 beats per minute is common and is considered normal. By measuring HR abnormalities, many types of cardiovascular diseases can be diagnosed [119].

Heart rate can be accurately measured by many techniques, ranging from electrical or optical to strain sensors. In term of electrical measurement, electrocardiography (ECG) monitors heart rate using electrodes. Because the ECG signals are periodic, the HR can be inferred from the R-wave-to-R wave (RR) interval of these periodic signals [107,108]. For example, Anliker et al. [107] investigated silver-coated chest suction electrodes (or adhesive silver/silver-chloride electrodes) without gel or paste and gold-plated electrodes as long-term ECG signal monitoring approaches. Xu et al. [109] used a pair of epidermal electrodes in a band aid form factor to monitor ECG signals from the sternum. The highlight of this approach is to use ideas from soft microfluidics, structured adhesive surfaces, and controlled mechanical buckling to achieve ultralow modulus, highly stretchable systems that incorporate assemblies of high-modulus, rigid, state-of-the-art functional elements. In addition, plethysmography is another powerful approach to measuring HR. When the heart beats, oxygenated blood is forced out of the heart while deoxygenated blood is pulled into the heart. This process distends the arteries and arterioles in subcutaneous tissue. Based on this theory, the HR can be detected by measuring the pressure of these subcutaneous tissues. For instance, Schwartz et al. [112] and Nie et al. [120] utilized high pressure sensitive flexible polymer transistors and droplet-based pressure sensors to achieve this pressure measurement. Moreover, sensitive magnetic sensors can also be used for quasi-noncontact pulse rate monitoring, such as amorphous metal wire-based magneto-impedance (MI) sensors, which are sensitive at pico-Tesla (pT) levels. These sensors have been shown to be able to measure a magnetocardiogram (MCG) in non-shielded conditions [121,122].

### 4.3. Respiration Rate

The human respiration (breathing) rate (RR) is another primary external physiological parameter that can indicate health status. Abnormal breathing rates suggest inefficient oxygen inhalation and carbon dioxide expulsion from the body’s tissues and are indicative of many diseases such as sleep apnea, asthma, chronic obstructive pulmonary disease, and anemia. Besides, RR monitoring is also one of important means for sleep monitoring [123]. To some extent, monitoring of sleep quality can be used to estimate the quality of health and even diagnosis of some disorders, such as sleep apnea, sudden death syndrome and heart diseases [124]. Typically, a healthy resting adult human RR is approximately one breath every 6.4 s, and the amount of air inhaled and exhaled is approximately 500 mL. A person’s RR tends to be constant across all ages. However, elderly people sometimes find it difficult to breathe normally. Internal lung structures and the respiratory system can change with old age, leading to breathing difficulties in elderly people. The rate of expansion and contraction of the lungs decreases, leading to more difficulty in breathing.

There are various approaches for long-term RR monitoring, but generally these can be categorized as either directly detecting airflow during the breathing process or indirectly responding to chest and abdomen expansion and contraction during breathing. For directly monitoring the breath flow, sensors can be located near the nose or mouth that responds to changes in air temperature, pressure, humidity, or carbon dioxide concentration as respiration occurs [110]. However, these sensors are not suitable for a smart clothing platform because they require inconvenient placement; consequently, this article does not discuss this approach further. The indirect method measures physical parameters such as detecting the changes in lung volume related to respiration. To date, various approaches have achieved measurements of electrical signal transduction and lung movement during inhalation and exhalation. With the rapid advance in textile-based technologies, a number of RR sensors have been built directly into textiles and are able to detect breathing rates accurately without reducing user comfort. For example, by integrating coated piezoresistive sensors in garments, Guo et al. [111] designed a wearable sensing system for long-term RR monitoring. Their system can distinguish the predominant breathing compartment (chest versus abdominal breathing) and is also capable of detecting a 10 s pause in breathing, which can be import in sleep apnea research. Another example was demonstrated referring to [125], where Atalay et al. developed a respiration belt using weft-knitted strain sensors to monitor RR. In this system, sliver-coated polymeric yarn was knitted together with elastomeric yarn to develop an interlocked knitted fabric with conductive loops in a single jersey arrangement. The sensing element is located within the fabric to avoid any contact between the human body and the sensing elements that could adversely affect the signal yield. For more information, please refer to [104].

### 4.4. Blood Pressure

Blood pressure (BP) measures the force of blood inside an artery. The two most significant numbers in blood pressure are the maxima (systolic) and minima (diastolic). Generally, the BP of a healthy person is regarded to be 120/80 millimeters of mercury (mm·Hg) where, the systole is 120 mm·Hg, and the diastole is 80 mm·Hg. Anything above 140/90 mm·Hg or below 120/80 mm·Hg is a matter for concern and should be checked. An increase (hypertension) or decrease (hypotension) of BP in the body indicate a malfunction. Both have many causes that can range from mild to severe and either may have a sudden onset or appear over long durations. Long term hypertension is a risk factor for many diseases, including heart disease, stroke and kidney failure. The reasons for changes in BP are still under investigation, but some causes include stress and being overweight. Increases in BP lead to other problems—especially heart problems. Changes in BP are typically not detrimental until approximately age 45 for both men and women; after which the adverse effects gradually tend to become more prominent.

Conventionally, BP is detected by using sphygmomanometers. However, these devices are not suitable for continuous healthcare systems because of their stationary setup requirements, cost, and lack of monitoring capability. The state-of-the-art sensor-based BP monitoring systems typically employ capacitive sensitive strain sensors [112], including both compressible and piezoelectric capacitive strain sensors. Compressible capacitive strain sensors are composed of an elastic, while piezoelectric capacitative strain sensors are composed of a robust dielectric sandwiched between two flexible electrodes. When the dielectric is squeezed by externally applied pressure, it will lead to the capacitance changes of the device. Similarly, if the piezoelectric material is strained, an induced voltage will be generated in the device. For example, Dagdeviren et al. [113] developed a conformable amplified lead zirconate titanate sensor with an enhanced piezoelectric response for cutaneous BP monitoring with a sensitivity and response time reaching approximately 0.005 Pa and 0.1 ms, respectively. This level of performance ensures that the sensor can be used to measure BP. In their BP measurement experiments, they attached this sensor to a subject’s wrist, arm or neck for long-term blood pressure monitoring. Their results suggest that these materials and the resulting sensor capabilities are feasible for BP monitoring. Additionally, RFID techniques have been shown to detect BP but they require device implantation under the skin [126].

### 4.5. Pulse Oxygenation

Oxygen saturation or oxygenation can be defined as the fraction of oxygen-saturated hemoglobin relative to total hemoglobin (unsaturated + saturated) in the blood. The human body needs to maintain a relatively precise and specific balance of blood oxygen. A 95%–100% blood oxygen level in human beings is considered normal. When this level falls below 90 percent, it is considered low, causing hypoxemia—particularly, tissue hypoxia—which is a major cause of morbidity and is ultimately the cause of death in most humans. According to the measurement location and method, oxygenation can be divided into three categories—tissue oxygenation (StO_2_), venous oxygenation (SvO_2_), and peripheral oxygenation (SpO_2_). Among all the different oxygenation measurement techniques, SpO_2_ measurement is ubiquitous because it is non-invasive.

Pulse oxygenation monitoring is typically achieved by monitoring SpO_2_ in a noninvasive way in fresh pulsatile arterial blood. The most frequently used measurement device is a pulse oximeter, which is an optics-based approach in which a pair of LEDs alternately shine light through a translucent part of the user’s body (usually a fingertip, earlobe, or area on the forehead or wrist). One LED is red, with a wavelength of 660 nm, and the other is infrared, with a wavelength of 940 nm. During a specific time, the intensity of light transmitted through the translucent part changes because of different levels of light absorption. More specifically, the blood volume and concentration of oxy-hemoglobin in the blood determine the extent of light absorption. A photodiode (PD) located at the opposite side is used to collect the transmitted light. Then, using a lookup table based on Beer-Lambert’s law, a pulse oxygenation measurement can be calculated [127]. In the past, the majority of commercially available products used inorganic optoelectronics that restricted sensing locations to finger tips or ear lobes due to their rigid forms and area-scaling complexity. Recently, with the advances in organic optoelectronics, the flexible form factors of organic LEDs (OLEDs) and organic photodetectors (OPDs) have become prime candidates for use in pulse oximetry because of their ability to conform to the human body [114]. An all-organic optoelectronic sensor system for pulse oximeter was presented in [114]. In this system, Lochner et al. [114] used green (wavelength: 532 nm) and red (wavelength: 626 nm) OLEDs coupled with an OPD, which are more compatible with flexible substrates, instead of a red and near-infrared LED pair. Compared with commercially available oximeters, the oxygenation measurement error of the all-organic sensor is below 2%. Additionally, another wearable organic optoelectronic sensor was presented in [115]. This sensor can be mounted on a forearm using a flexible bandage that incorporates the photodiodes and an OLED light source in the middle. The results of experiments successfully showed changes in the tissue concentration of oxy-hemoglobin upon induction and termination of ischemia induced in the arm.

### 4.6. Blood Glucose

Glucose is commonly considered to be the primary source of energy for human cells. From a physiological aspect, glucose is delivered from the intestines or liver to body cells via the bloodstream and is made available for cell absorption through the hormone insulin, which is produced primarily in the pancreas. Blood glucose measurements reflect the amount of glucose in human blood. Its concentration is usually lowest in the morning and increases after meals. A blood glucose measurement out of its normal range may indicate the need for medical care. Commonly, hyperglycemia is indicated by a continuously high blood glucose level while hypoglycemia is indicated from a low level. Diabetes is caused by persistent hyperglycemia and is the most common diseases related to abnormal blood glucose regulation. The World Health Organization reports that 9% of adults worldwide suffer from diabetes. Therefore, daily blood glucose monitoring is essential both for preventing diabetes and improving the health and quality of life of people who suffer from diabetes.

The approaches to blood glucose measurement can be divided into two types: those that require blood and those that do not [103]. Considering wearable smart clothing scenarios, the methods that require blood are unsuitable for real-time monitoring systems because procedures to obtain blood are invasive and inconvenient, require patient compliance, and the blood sampling processes risk infection [103]. In this article, we mainly review the techniques that do not require drawing blood. Generally, the noninvasive blood glucose measurement sensors detect the glucose concentration using an enzyme-based electrochemical sensor. The major enzyme used for this detection is glucose oxidase (GOD). The principle is presented in [103]. Tierney et al. [128] introduced an electrochemical glucose sensor for noninvasive glucose monitoring. Based on the reverse iontophoresis technique, skin interstitial fluid (ISF) glucose is drawn to the skin surface and then detected by an enzymatic electrochemical glucose sensor. The reverse iontophoresis process applies a mild electrical current to the epidermis, causing ions to migrate through the skin toward the electrodes. Sodium ions compose the majority of charge carriers because the skin has a neutral pH. The migration of sodium ions from across the skin to the cathode leads to an electro-osmotic flow of ISF toward the cathode. This flow also transports glucose toward the cathode. Consequently, this technique can obtain glucose from blood. Another noninvasive approach is described in [116], where Liao et al. present a contact-lens-mounted glucose biosensor platform for continuous heath monitoring. This sensor system integrates a loop antenna, a wireless sensor interface chip, and a glucose sensor on a polymer substrate. The glucose sensor uses tear fluid, which contains glucose in the range of 0.1–0.6 millimoles per liter (mM)—A level approximately ten times lower than blood level. Their system achieves a sensitivity of 400 Hz/mM while consuming 3 W from a regulated 1.2-V supply. For more blood glucose measurement approaches, please refer to [117].

## 5. Discussion

Because it is of great importance that we can find the elderly person timely when risky circumstances occur, so that a prompt response from doctors and nurses to avoid additional injuries. Thus the positioning technologies is equal importance as vital sign monitoring and physical activity recognition in the elderly care scenarios. In this review, compared with the current concept of smart clothing/clothes that emphasizes on vital sign monitoring and physical activity recognition, we add the function of positioning to extend this concept so that the systems or platforms we proposed can meet the specially demands of elderly care.

### 5.1. Positioning Technologies

Positioning technologies are divided into two categories: outdoor positioning and indoor positioning. The proper and practical method to realize the outdoor positioning is using commercial GPS. So choosing a suitable indoor positioning scheme for elderly care is more problematic. From the above reviewing, some existing technologies, such as UWB, RFID, visible Light, can meet the requirement of healthcare scenarios. However, these technologies need to prearrange necessary devices as base stations. To construct these positioning networks is costly because many base stations is needed to cover complex daily living environment of elderly people. Besides, they cannot position in unreached area such as the elderly go shopping in supermarket. The indoor location schemes using geomagnetism or motion sensors (an integration of a three-axis gyroscope, three-axis magnetometer, and three-axis accelerometer) seem to be suitable for the elderly care scenarios because of low-cost, no extra devices, and can serve to position at unpredicted areas. But, the accuracies of geomagnetic IPS or PDR systems (which range from 0.1 m to 2 m and from 1 m to 5 m, respectively) are not precise enough to meet the demands of AAL in elderly care scenarios. Therefore, a supplementary approach must be adopted to achieve a robust and precise indoor positioning and tracking system. For example, the authors of [28,44,45,47,129,130,131,132,133,134,135] combined PDR with other approaches to improve localization accuracy (e.g., GPS, ultrasound ranging, active RFID, WiFi signatures, and Chirp Spread Spectrum (CSS) radio beacons). The accuracy of these approaches can be greatly improved compared with stand-alone PDR systems, with errors reaching below 1.7 m. Hence, in this review, a scheme fusing a PDR system with a magnetic indoor positioning technique is recommended. For one reasons, this fusing can be used to reduce the localization error; for another reason, individual PDR or geomagnetic IPS can work in the low-accuracy scenarios such as semi-outdoor.

### 5.2. Physical Activity Detection

Human activity detection and monitoring during daily life is another significant function for elderly care. With continuous and timely activity monitoring, elderly people can benefit from effective actions taken when abnormal situations or unpredictable events occur. Sensor-based HAR has benefited from the rapid development of MEMS sensor technologies. Thereinto, accelerometers have been employed in the bulk of sensor-based HAR applications so far. However, these systems have limitations. HAR systems that rely solely on accelerometers do not perform well in some complex activity recognition scenarios because an accelerometer provides only acceleration information. Consequently, sensors such as gyroscopes, magnetometers, and barometric pressure sensors have been combined with accelerometers to improve the performance of complex activity recognition. Sensor placements are determined by the type of activities to be recognized. As classification algorithms, criterions, such as recognition accuracy, power-consumption, and the practicality of the entire systems, should be taken into consideration. Algorithms that consume more power will reduce or restrict the operational duration. Thus, HAR algorithms for “smart clothing” have carefully considered how to remain computationally light-weight. In terms of the type of sensors, the hybrid accelerometer, gyroscope and magnetometers is recommended to construct elderly care systems, because complex activities are also needed to be recognized and tracked during the daily lives of the elderly. With the repaid development of different computational units, such as GPUs, low-power CPU cores, multi-core CPUs, coupled with increasing amounts of memory, it have become possible to use complex models like DL algorithm to improve the performance of HAR systems, both accuracy and activity category. Hence, elderly care systems that employ this much more intelligent algorithm will become the future trends.

### 5.3. Vital Sign Monitoring

Due to aging, most elderly people are obsessed with various age-related diseases. Therefore, through real-time monitoring of health parameters, some pathemas can be prevented in non-clinical environments instead of being treated in hospitals. Regular monitoring of vital signs allows the construction of an individual's health baseline and can alert both users and medical professionals of risky situations that may require further medical attention. This review chiefly summarized and discussed flexible, noninvasive and wearable vital-signs monitoring sensors, focusing on bio-signals such as temperature, heart rate, respiration rate, blood pressure, pulse oxygenation. These wearable sensors composed of flexible and stretchable materials have the potential to better interface to the human skin. From the data processing and transmission point of view, silicon-based electronics are extremely efficient, which can be used to construct a monitoring and alarming systems. If these flexible and stretchable sensors combine with low-power electronics, these systems can consume less power and work with a long duration to support wide coverage and mobility. In the long run, these biosensors will become tinier, smarter, and more energy conservation. Another important aspect of vital sign monitoring is how the collected bio-data are fused and processed for prediction, diagnosis, decision making and guidance to lead a healthy lifestyle (note that functions, such as eating behavior quantification, exercising properly, eating healthy diet, etc., are the vital future use trends of vital sign monitoring.) The proper method is to constitute wearable Body Area Networks (BANs). In the future, these BANs may be based on nanonetworks, a completely novel communications paradigm that aims at copying natural molecular and cell communications [136].

## 6. Conclusions

This article provided an overview of the state-of-the-art technologies for designing smart clothing platforms, focusing mainly on accurate indoor positioning, HAR, and vital sign monitoring. For outdoor positioning commercial products based on the GPS, GLONASS, or BDS are adequate; for indoor scenarios, systems that use a fusion of magnetic IPS and PDR were analyzed and recommended. For HAR, the available technologies can be divided into three categories: vision-based recognition, radio-based recognition, and sensor-based recognition. Sensor-based technologies were recommended for elderly care, particularly combinations that involve accelerometers and gyroscopes. In addition, we also provided an overview of the classification algorithms commonly used in sensor-based systems. For vital sign monitoring, the discussion was limited to only wearable and non-invasive technologies. Six vital signs routinely monitored by medical professionals were included. We hope that this review will provide readers with the underlying principle of the “extended smart clothing platform” for elderly care. We also hope this review will convince readers that with further improvement of wearable technologies coupled with low-cost and large-area processing capabilities, the promise of applications for elderly care is enormous.

## Figures and Tables

**Table 1:** Comparison of indoor positioning technologies.

Technology	Measurement Methods	Suitable Environment	Accuracy	Extra Device on User-side	Power Consumption	Cost	Advantages	Disadvantages	Examples
A-GPS ^1^	TTFF ^2^	Outdoor and Indoor	5–10 m	No	High	Low	Reuse sensors embedded in smartphone or smartwatch; cover entire earth.	Low security; occupy channel resource.	Google Earth; Baidu Maps
GSM (cellular)	RSS ^3^	Outdoor and indoor	10–50 m	No	Low	Low	Free of same-frequency interference; reuse sensors embedded in smartphone or smartwatch [33].	Low reliability [33]; privacy issues.	Google Maps
RFID	Proximity; RSS	Indoor	1–3 m	Yes	Low	Moderate	Moderate cost; high accuracy.	Tags lack communications capabilities; positioning coverage is limitted; extral devices.	Cricket (MIT) [37] SpotON (UW) [38] RADAR(Microsoft) [39]
WiFi	RSS	Indoor	1–5 m	No	High	Low	Reuse existing infrastructure; low infrastructure cost.	Fingerprinting systems recalculation [33].	Nibble [40] Wayes [41]
UWB	ToA; TDOA	Indoor	6–10 cm	Yes	Low	High	Excellent accuracy; effectively passing through obstacles.	High cost; short range; problem in non-Line of Sight.	Ubisense; Dart UWB(Zebra).
Dead Reckoning	Tracking	Indoor or Outdoor	1–5 m	No	High	Low	No additional hardware such as beacons.	Low accuracy.	/
Infrared	Proximity; Differential Phase-shift; AoA ^4^.	Indoor	1–2 m	Yes	Low	Moderate	Low power consumption.	Short rang; cost for extra hardware.	IR.Loc (Ambiplex) [42]
BLE Beacon	Proximity; RSS	Indoor and Semi-outdoor	1–5 m	No	Low	Low	Low infrustructure cost; low power consumption.	Limitation in user mobility; low accuracy.	Shopstic (App).
Acoustic Signal	ToA; TDOA	Indoor	0.03–0.8 m	No	Low	Moderate	No requirement for line of sight (LOS); does not interfere with electromagnetic waves [32].	Cannot penetrate solid walls; loss of signal due to obstruction; false signals because of reflections [32].	Active Bat; Sonitor IPS.
ZigBee	RSS	Indoor	1–10 m	No	Low	Low	Low infrastructure cost; low power consumption; short time delay; high security.	Short range	Best Beacon Match [43]
Visible Light	ToA; TdoA ^5^.	Indoor	0.01–0.1 m	Yes	Low	High	Dual use of lighting infrastructure; compatible with RF sensitive areas.	Needs to replace existing lights to LEDs ^6^ (dual use); High cost	Bytelight; Ubeacon.
Image-Based IPS	Pattern recognition	Indoor	0.01–1 m	No	High	Moderate	Relatively cheap compared with technologies such as UWB.	Requires LOS, coverage is limited	Sky-Trax; StarGazer.
Geomagnetism-based IPS	Maps matching	Indoor and Outdoor	0.1–2 m	No	Low	Low	No requirement of the maintenance (reusing existing device); between sensor and source; the ability to penetrate walls.	Interference by environment magnetic fields.	IndoorAtlas (University of Oulu)

^1^ A-GPS: Assisted GPS [34]; ^2^ TTFF: time-to-first-fix [35]; ^3^ RSS: received signal strength; ^4^ AoA: angle of arrival; ^5^ ToA: time of arrival; TDoA: time difference of arrival; ^6^ LED: light-emitting diode.

**Table 2:** Summary of requirements for the elderly care system.

Criterion	Description	Value
Accuracy	2D position compared to reference	0.5–1 m
Installation complexity	The time to install an IPS in a flat	<1 h
User acceptance	A qualitative measure of invasiveness	Non-invasive
Coverage	Area of a typical living flat	90 m^2^
Update rate	The sampling interval of an IPS	0.5 s
Operating time	The battery life	Not assessed
Availability	The time that a system is active and responsive	>90%

**Table 3:** Summary of research on sensor placement for HAR.

Sensor	Location	Activities	Reference
Gyroscope Accelerometer	Wrist, hip, neck, knee cap	Wing Tsun movements	Heinz et al. [13]
Accelerometer	Ankle, thigh, hip, wrist, chest	Typing, talking, riding, walking, arm movement, etc. (20 activities)	Bao et al. [74]
Accelerometer	Thigh, Necklace, Wrists.	Falling backward, falling forward, chest pain, headache, vomiting, and fainting and a normal activity walking	Pirttikangas et al. [59]
Accelerometer	Waist.	Walking, running, scrubbing, standing, working at a PC, vacuuming, brushing teeth, sitting.	Yang et al. [71]
Accelerometer, Gyroscope	Lower arm, Hip, Thigh, Wrist	Walking downstairs, walking upstairs, walking, jogging, biking, sitting and standing.	Shoaib et al. [66]
Accelerometer	Thigh	Walking, jogging, ascending stairs, descending stairs, sitting, standing.	Kwapisz et al. [75]
Accelerometer	Lower Back.	Lying, sitting, standing, working. on a computer, walking, running, cycling.	Bonomi et al. [72]
Accelerometer	Hip, wrist, arm, ankle, thigh	Lying, sitting, standing, walking, stair climbing, running, cycling.	Mannini et al. [58]
Accelerometer; gyroscope	Upper arm, thigh	Slow walking, normal walking, brisk walking, jogging, sitting, ascending and descending stairs normally or briskly	Wu et al. [60]
Accelerometer	Chest, thigh, ankle.	Stairs ascent and descent, walking, sitting, standing up, sitting on the ground	Chamroukhi et al. [69]
Accelerometer	Chest, thigh, ankle.	16 daily living activities.	Moncada-Torres, et al. [68]
Accelerometer gyroscope	Thigh	Walking, walking upstairs, walking downstairs, sitting, standing, and lying down	Ronao et al. [76]
Accelerometer; Gyroscope; Barometric pressure sensors.	Wrist; ankle; chest	Walking, running, stair descending and ascending, standing, sitting, lying down, brushing teeth, drinking, cutting food, writing, peeling carrot, eating butter bread, etc.	Moncada-Torres, et al. [68]

**Table 4:** Summary of features for pre-processing [77].

Group	Method
Time domain	Mean, median, standard deviation, variance, minimum, maximum, range, root mean square (RMS), correction, cross-correlation, entropy, and kurtosis, skewness, peak to peak, crest factor [56], difference, zero crossing, integration, mean absolute deviation (MAD) etc.
Frequency domain	Fourier transform (FT), coefficients sum, dominant frequency, spectral energy, peak frequency, information entropy, entropy spectrum, spectral analysis of key coefficients, frequency range power (FRP) [13], etc.

**Table 5:** State of the art of human activity classification systems.

Sensors	Placement	Features	Classifiers	Participants	Activities	Accuracy (%)	Reference
Accelerometer	Upper arm, lower arm, hip, thigh, foot	Time-domain; frequency-domain.	KNN; Decision tree; NB.	20	6	52–84	Bao. et al. [74]
Gyroscope	Shank	Frequency-domain	Other	20	1	97	Coley et al. [61]
Accelerometer; gyroscope.	Wrist; lower leg; foot; neck; hip.	Time-domain; frequency-domain.	Decision tree; KNN; NB.	2	20	NA ^1^	Heinz et al. [13]
Accelerometer	Wrist; hip; necklace	Time-domain	C4.5 ^2^	6	6	About 75	Muurer et al. [83]
Accelerometer	Lower back	Time-domain; frequency-domain.	Decision tree	20	20	93	Bonomi et al. [72]
Accelerometer; gyroscope.	Chest, thigh	Time-domain	User-defined	1	4	91	Li et al. [63]
Accelerometer	Wrist; lower arm; knee; ankle.	Time-domain; frequency-domain.	NB, GMM, SVM, NV, C4.5	20	20	92.2–98.5	Mannini et al. [58]
Accelerometer	Thigh	Time-domain; frequency-domain.	C4.5, MLP ^3^, LR ^4^	29	6	78.1–95.7	Kwapisz et al. [75]
Accelerometer; gyroscope	Arm, thigh.	Time-domain; frequency-domain.	KNN; C4.5; NB, etc.	16	13	63.2–90.2	Wu et al. [60]
Accelerometer; gyroscope	Lower arm	Time-domain	RBM ^5^	12	NA	72.1	Bhattacharya et al. [92]
Accelerometer; gyroscope	Wrist	Time-domain; frequency-domain.	HF-SVM ^6^	30	6	89	Anguita et al. [93]
Accelerometer	Lower back	Time-domain	Decision tree	24	4	96.61	Khan et al. [94]
Accelerometer; gyroscope; barometric pressure sensors	Ankle; wrist; chest	Time-domain; frequency-domain	KNN	6	16	93–95	Moncada-Torres et al. [68]
Accelerometer	Wrist	Time-domain; frequency-domain	NB; SVM; Decision tree; KNN	2	8	57–64	Ravi et al. [86]
Accelerometer	Chest; upper arm; wrist; hip; thigh; ankle; ear.	Time-domain; frequency-domain	KNN; Bayesian	11	15	NA	Atallah et al. [85]
Accelerometer.	Thigh	Time-domain	Shapelet approach; SVM; NB; KNN; etc.	4	8	72–77	Liu et al. [57]
Accelerometer; gyroscope	Wrist	Time-domain	ANN; SVM; NB; C4.5	30	6	76.63–95.75	Ronao et al. [76]
Accelerometer; gyroscope.	Thigh	Time-domain	RF; SVM; NB.	NA	4	77–99	Bedogni et al. [95]
Accelerometer; Gyroscope.	Chest, thigh, ankle	Time-domain; frequency-domain.	Decision tree; KNN; SVM; HMM, etc.	6	12	73–98	Attal et al. [56]
Accelerometer	Wrist	Time-domain;	NB; Decision tree; SVM; C4.5; KNN	10	6	63.09–99.56	Morillo et al. [96]
Accelerometer	Wrist	Time-domain; frequency-domain.	NN; KNN^.^	13	6	79.2–90.4	Wang et al. [87]
Accelerometer	Hip	Time-domain; frequency-domain.	KNN; SVM.	5	9	70–75.68	Wang et al. [97]

^1^ NA: Not Available; ^2^ C4.5: Decision trees; ^3^ MLP: Multilayer perception; ^4^ LR: Logistic Regression; ^5^ RBM: Restricted Boltzmann Machine; ^6^ HF-SVM: Hardware-Friendly SVM.

**Table 6:** Summary of several vital signs and measurement technologies.

Vital Sign	Range & Scale	Technique	Tranduced Signal	References
Body temperature	32–45 °C	Thermistors; thermoelectric effects; optical means	Resistance	Husain et al. [104]; Chen et al. [105]; Richmond et al. [106].
Heart rate	0.5–4 mV (ECG)	Skin electrode; optical; MI sensor.	Voltage/Current	Anliker et al. [107]; Rienzo et al. [108]; Xu et al. [109].
Respiration Rate	2–50 b/min ^1^	Strain gauge/Impedance	Resistance	Folke et al. [110]; Guo et al. [111].
Blood pressure	10–400 mm Hg	Piezoelectric capacitors; capacitive strain sensors	Drain current	Schwartzet al. [112]; Dagdeviren et al. [113]
Pulse oxygenation	80%–100% (SpO_2_)	Optical means.	Photodiode current	Lochner et al. [114]; Bansal et al. [115]
Blood glucose	0.5–1 mM ^2^	Electrochemical	Current	Liao et al. [116]; Vashist [117]

^1^ b/min: breaths/min; ^2^ mM: millimoles per liter.
